# Small bowel obstruction secondary to a migrated biliary stent encased in a fecaloma: a case report

**DOI:** 10.1093/jscr/rjag1024

**Published:** 2026-11-10

**Authors:** Shaikha Alshamsi, Marwan Faris, Omar Alsayed, Syed M Javed, Shamma Al Aleeli, Rashad Mohammed

**Affiliations:** Department of General Surgery, Dubai Hospital, Al Baraha, Dubai, United Arab Emirates; Department of General Surgery, Dubai Hospital, Al Baraha, Dubai, United Arab Emirates; Department of General Surgery, Dubai Hospital, Al Baraha, Dubai, United Arab Emirates; Department of General Surgery, Dubai Hospital, Al Baraha, Dubai, United Arab Emirates; Department of General Surgery, Dubai Hospital, Al Baraha, Dubai, United Arab Emirates; Department of General Surgery, Dubai Hospital, Al Baraha, Dubai, United Arab Emirates

**Keywords:** case report, small bowel obstruction, biliary stent migration, fecaloma

## Abstract

Distal migration of biliary stents is a recognized complication of biliary drainage, although small bowel obstruction secondary to a migrated stent is rare. We report the case of a 29-year-old male who presented with acute small bowel obstruction 6 years after biliary stent placement. Computed tomography demonstrated a distal small bowel obstruction and a tubular intraluminal structure suspicious for a migrated stent. Colonoscopy identified a large impacted fecaloma, but endoscopic retrieval was unsuccessful. Exploratory laparotomy revealed a migrated biliary stent completely encased within a giant fecaloma, which was removed through a limited enterotomy while preserving bowel viability. This case highlights an unusual delayed mechanism of stent-related obstruction and the role of surgery when endoscopic retrieval is unsuccessful.

## Introduction

Biliary stents are commonly used to maintain biliary drainage in benign and malignant biliary disorders. Although migration is a recognized complication, most migrated stents pass through the gastrointestinal tract without significant consequences [1]. Small bowel obstruction secondary to stent migration is uncommon and is described predominantly in isolated case reports [3–8]. Delayed presentation may complicate diagnosis, particularly when the original biliary intervention occurred several years earlier. We present a rare case of small bowel obstruction caused by biliary stents placed 6 years previously that subsequently migrated and became encased within a large fecaloma in the distal ileum.

## Case report

A 29-year-old Sudanese male presented to the emergency department with a one-day history of suprapubic abdominal pain and three episodes of non-bloody, non-bilious vomiting. He subsequently developed abdominal distension and an inability to pass flatus. Otherwise, he denied fever, dysuria, previous similar episodes, or a preceding history of constipation.

His past medical history was unremarkable. Six years earlier, laparoscopic cholecystectomy at another institution was converted to open surgery because of intraoperative bleeding. Bile duct injury and leakage subsequently required exploratory laparotomy and hepaticojejunostomy. Approximately 6 months later, he underwent endoscopic retrograde cholangiopancreatography with biliary stent placement, followed by another laparotomy for postoperative intra-abdominal collections. Details of the stents, intended duration, and planned removal or follow-up were unavailable.

On presentation, blood pressure was 98/62 mmHg, heart rate 97 beats/min, and temperature 36.8°C. The abdomen was initially tender suprapubically without peritonitis but became progressively distended and diffusely tender. Initial investigations showed a white blood cell count of 7.1 × 10^3^/μl, C-reactive protein of 2.5 mg/l, lactate of 0.9 mmol/l, and normal renal and liver function.

Contrast-enhanced computed tomography (CT) of the abdomen and pelvis demonstrated dilated small bowel loops with fecalization and distal ileal narrowing, consistent with partial small bowel obstruction. A tubular, hyperdense intraluminal structure was seen distally in the small bowel, raising suspicion for a migrated biliary stent (Fig. 1).

**Figure 1:**
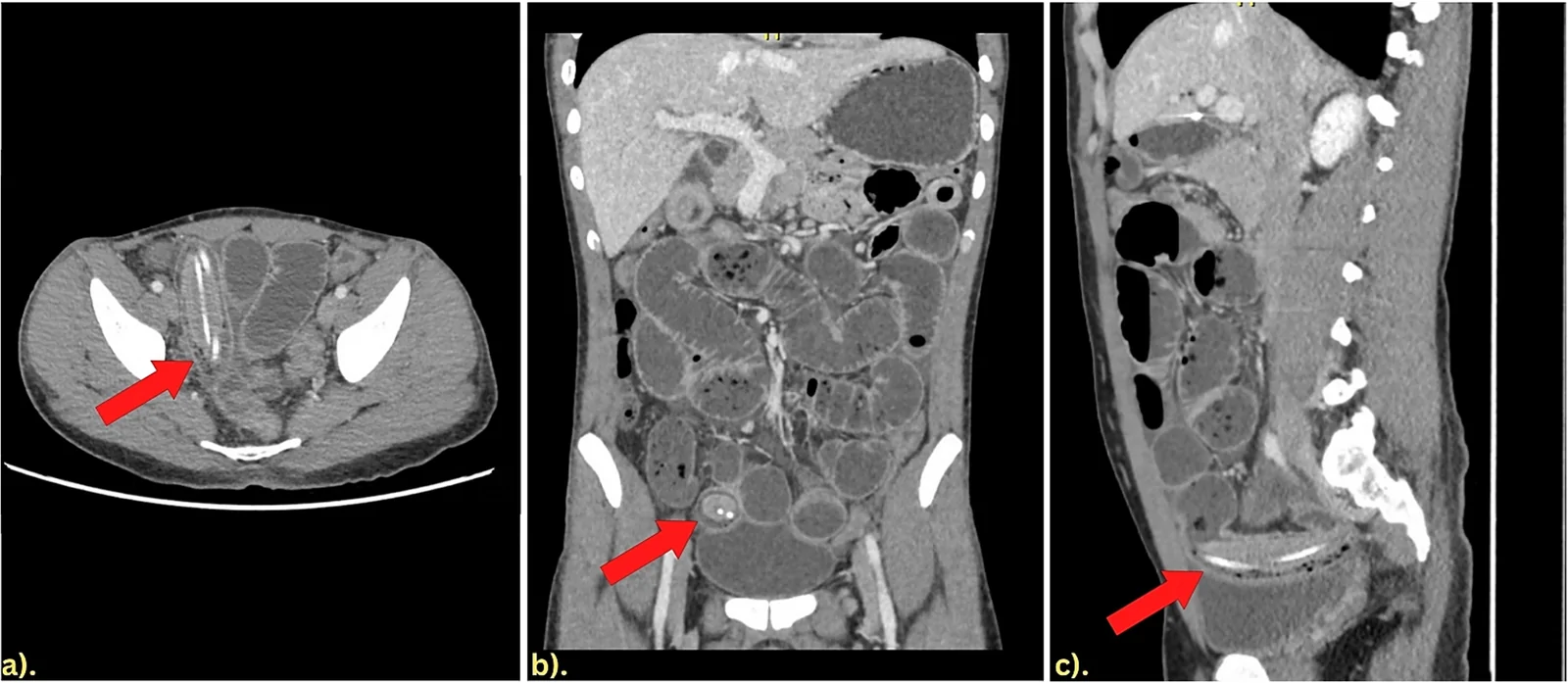
Contrast-enhanced CT images demonstrate small bowel obstruction caused by a migrated biliary stent encased within a fecaloma. The arrows indicate the fecaloma-encased stent. (a) Axial image. (b) Coronal image showing the stent-containing fecaloma in the right iliac fossa, with proximal small bowel dilatation and fecalization. (c) Sagittal image showing the stent-containing fecaloma and dilated small bowel loops.

Initial management included bowel rest, nasogastric decompression, intravenous fluids, antibiotics, analgesia, and antiemetics. Colonoscopy demonstrated a large, hard fecaloma measuring ~7–8 cm within the ileum (Fig. 2). Attempts at retrieval with a cold snare, grasping forceps, and irrigation were unsuccessful, prompting operative removal.

**Figure 2:**
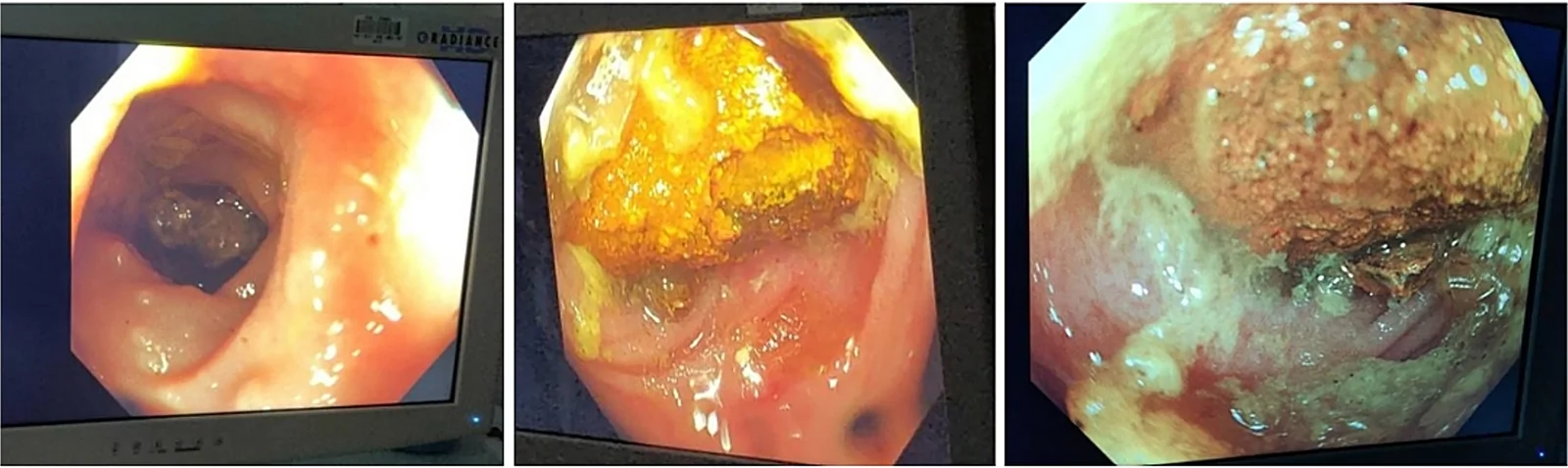
Colonoscopic images demonstrating the hard fecaloma impacted within the ileum.

At exploratory laparotomy, a large intraluminal obstructing mass was identified ~60–70 cm proximal to the ileocecal junction. Following proximal milking, a 1-cm antimesenteric enterotomy revealed migrated biliary stent material completely encased within a large, hardened fecaloma (Fig. 3). The mass was extracted and the enterotomy was closed primarily. The involved bowel was viable, without ischemia, perforation, or serosal injury.

**Figure 3:**
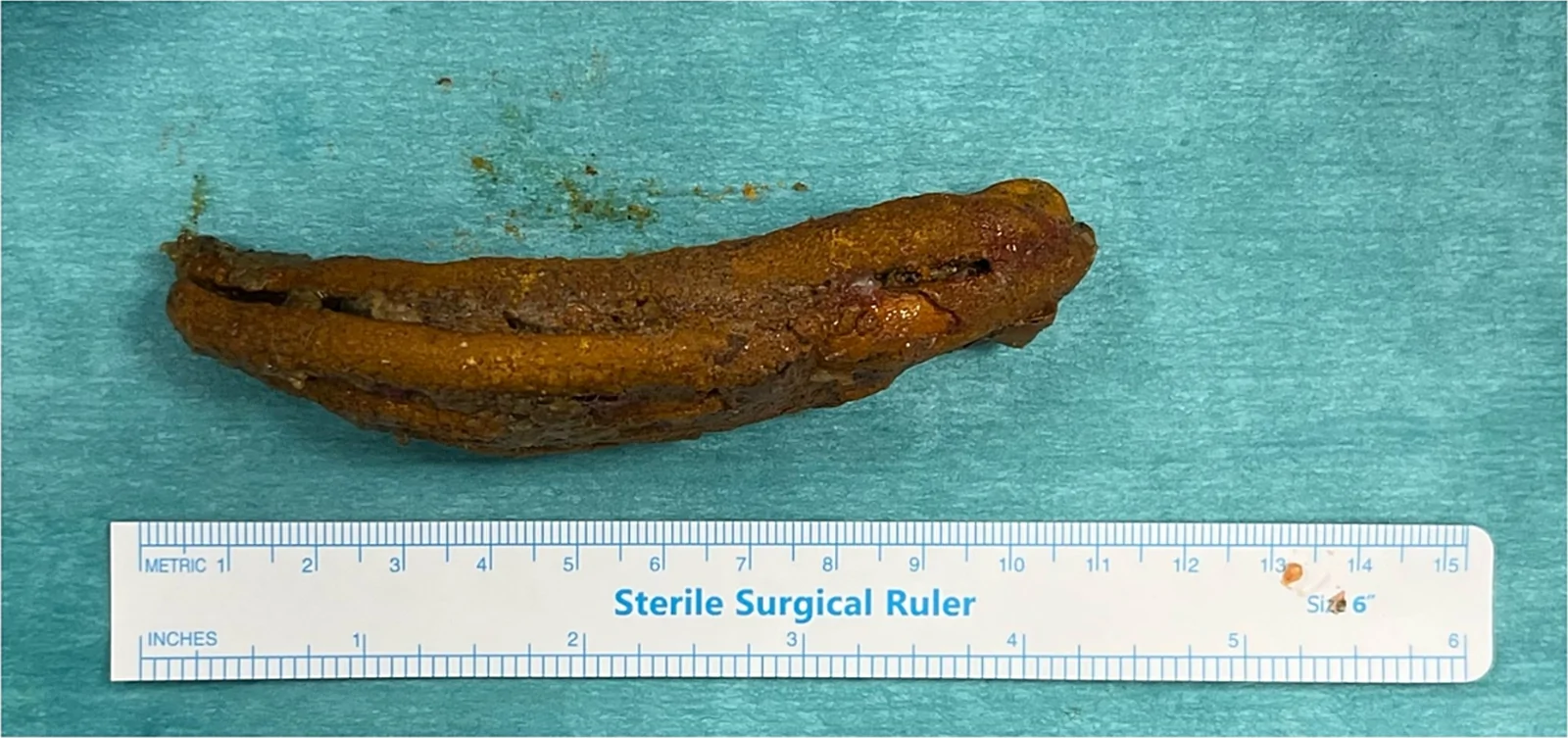
Biliary stent completely encased within a large, hardened fecaloma following enterotomy.

Pathological examination identified two separate pieces of foreign material consistent with biliary stents, measuring 11.0 × 2.0 × 2.0 cm and 11.0 × 0.5 × 0.5 cm, respectively, surrounded by stony-hard fecal material consistent with a fecaloma.

Postoperatively, transient hypotension responded to intravenous fluids without vasopressor support. He subsequently remained stable, bowel function returned, and diet was advanced as tolerated. No wound infection, intra-abdominal collection, or recurrent obstruction occurred. He was discharged home on postoperative day 9, with outpatient follow-up with General Surgery arranged.

## Discussion

Biliary stent migration is a recognized complication of biliary drainage procedures. In a retrospective cohort study of 876 patients who underwent biliary stent placement, migration occurred in 74 patients (8.4%), with distal migration (5.5%) occurring more frequently than proximal migration (3.0%). Independent predictors of migration included moderate-to-marked common bile duct dilatation, complete endoscopic sphincterotomy, biliary balloon dilatation, and an indwelling stent duration exceeding 1 month [1]. Although most distally migrated biliary stents traverse the gastrointestinal tract uneventfully, impaction or penetration may result in intestinal obstruction, perforation, bleeding, fistula formation, or infection [1, 2]. A systematic review of bowel perforation following biliary stent migration identified 132 cases, more than half requiring surgical management. However, as this review specifically evaluated perforation, its reported outcomes should not be extrapolated to small bowel obstruction caused by migrated biliary stents [2].

Small bowel obstruction caused by migrated biliary stents remains rare and is described predominantly in isolated case reports. Reported mechanisms include direct ileocecal or jejunal impaction, adhesion-related luminal narrowing, bowel-wall embedding, and secondary concretion formation, with management ranging from conservative treatment to colonoscopic retrieval, laparoscopic extraction, enterotomy, and bowel resection [3–8].

Rasalkar *et al.* described ileocecal obstruction successfully treated by colonoscopic retrieval [3], while Garg *et al.* managed a jejunal obstruction with combined laparoscopic and endoscopic extraction without enterotomy [4]. Golfeyz *et al.* described adhesion-related obstruction that resolved following further distal stent migration [5]. Conversely, Nudelman *et al.* and Ghirardelli *et al.* reported ileal obstruction requiring bowel resection [6, 7]. Similarly, Rumble *et al.* described a jejunal impaction at an intra-loop adhesion, requiring laparotomy and enterotomy with primary closure [8].

The present case is notable for the 6-year interval after stent placement and its mechanism of obstruction: two migrated stents were encased within a large hardened fecaloma impacted 60–70 cm proximal to the ileocecal junction. Ghirardelli *et al.* similarly described obstruction associated with secondary lithiasic concretion around a migrated stent [7]. However, complete fecaloma encasement represents a distinct mechanism of secondary obstruction. The patient’s history of multiple abdominal operations may also have contributed to impaired stent transit and eventual impaction, as postoperative adhesions have been implicated in previous reports [5–8]. Nevertheless, the immediate obstruction was caused by the fecaloma containing the stents, and the timing of migration and fecaloma formation remains uncertain.

Management should be individualized according to the anatomical location and accessibility of the migrated stent, degree of impaction, bowel-wall embedding, presence of secondary obstructing material, evidence of ischemia or perforation, and feasibility of endoscopic retrieval [3–8].

In this case, colonoscopic retrieval failed because of the size and firmness of the fecaloma. Limited antimesenteric enterotomy allowed removal while preserving viable bowel and avoiding resection. This case highlights an unusual delayed, fecaloma-associated mechanism of obstruction and demonstrates that, when endoscopic retrieval fails and bowel viability is preserved, enterotomy may provide an effective bowel-preserving option.
